# Comparative Karyotype Analysis of Parasitoid Hymenoptera (Insecta): Major Approaches, Techniques, and Results

**DOI:** 10.3390/genes13050751

**Published:** 2022-04-25

**Authors:** Vladimir E. Gokhman

**Affiliations:** Botanical Garden, Moscow State University, 119234 Moscow, Russia; vegokhman@hotmail.com; Tel.: +7-495-939-3293

**Keywords:** chromosomes, cytogenetics, karyotypes, parasitic wasps, parasitoid Hymenoptera

## Abstract

A comprehensive review of main approaches, techniques and results of the chromosome study of parasitic wasps is given. In this group, the haploid chromosome number ranges from *n* = 3 to 23. Distribution of parasitic wasp species by the chromosome number is bimodal, with two obvious modes at *n* = 6 and 11. Karyotype analysis based on routinely stained preparations of mitotic chromosomes can be used to identify members of taxonomically complicated parasitoid taxa and to distinguish between them. Morphometric study effectively reveals subtle differences between similar chromosome sets of parasitic wasps. If combined with meiotic analysis and/or cytometric data, information on mitotic karyotypes can highlight pathways of the genome evolution in certain parasitoid taxa. C- and AgNOR-banding as well as staining with base-specific fluorochromes detected important interspecific differences within several groups of parasitic wasps. Fluorescence in situ hybridization (FISH) is successfully used for physical mapping of various DNA sequences on parasitoid chromosomes. These techniques demonstrate that heterochromatic segments are usually restricted to pericentromeric regions of chromosomes of parasitic wasps. Haploid karyotypes carrying one or two nucleolus organizing regions (NORs) are the most frequent among parasitoid Hymenoptera. In combination with chromosome microdissection, FISH could become a powerful tool exploring the genome evolution of parasitic wasps. Perspectives of the comparative cytogenetic study of parasitoid Hymenoptera are outlined.

## 1. Introduction

The order Hymenoptera is one of the most species-rich, taxonomically complicated and economically important groups of insects, which contains more than 150 thousand described species [1]. At present, this order is subdivided into two suborders, Symphyta and Apocrita [2]. The latter suborder contains two traditional major groups, i.e., Parasitica and Aculeata, or parasitic and aculeate wasps, respectively [3]. In turn, parasitoid Hymenoptera harbor a few superfamilies, e.g., Ichneumonoidea, or ichneumonoid wasps (with the families Ichneumonidae and Braconidae), Cynipoidea, or gall wasps (includes five families, i.e., Cynipidae, Figitidae etc.), and Chalcidoidea, or chalcid wasps (with more than 20 families, e.g., Aphelinidae, Encyrtidae, Eulophidae, Eupelmidae, Eurytomidae, Pteromalidae, Torymidae, and Trichogrammatidae) [1].

Despite there being currently about 80 thousand described species in the world fauna of parasitoid Hymenoptera [1], their potential number certainly exceeds one million [4,5]. Many parasitic wasps are of considerable economic importance, since they attack a wide array of insects and other arthropods, including a number of significant pests of agriculture and forestry [6]. However, cytogenetic features of parasitoid Hymenoptera remain poorly studied. Specifically, chromosomes of about 500 species of this group are known up to now [7], which therefore constitutes less than 0.05 per cent of their potential number. Nevertheless, certain important studies in this field were published during recent decades. These works investigated parasitoid karyotypes using both routine chromosome staining as well as more advanced techniques (see below). In turn, this research had various aims, which defined the whole range of different approaches, methods and results obtained during these studies. In particular, data obtained by modern techniques are predominantly used in genetic studies of parasitic wasps [8]. On the other hand, information on the chromosome number and analogous features of the karyotype structure is mostly, but not exclusively, requested by contemporary taxonomy of this group [9]. Moreover, chromosomal characters can successfully distinguish between closely related species of parasitoid Hymenoptera, including cryptic ones (see below). The present survey therefore reviews the current state of the karyotypic study of parasitic wasps with an emphasis on the last 10–15 years.

## 2. Techniques Used

Historically, chromosomes of Hymenoptera were initially studied on sectioned material [10]. However, sectioning was an inappropriate method of the karyotypic study, since it led to serious errors in evaluating chromosome number, shape and size [11]. Later on, the technique of squash preparations was introduced into the chromosome study of hymenopterous insects [10]. This method provides better results in terms of chromosome morphology, but the preparations obtained cannot be stored for a long time; nevertheless, they can be converted into permanent ones using dry ice or liquid nitrogen. Although the technique of squash preparations was used for studying parasitoid karyotypes for several decades [12,13,14,15], an important improvement of this method was implemented in the 1960s. This modification included pretreatment of the tissue in question with colchicine (or colcemid) and hypotonic saline solution to block the dividing cells at metaphase and to make the chromosomes spread apart [16]. After that, the tissue was treated with, e.g., 60% acetic acid to turn it into cell suspension, which was then applied to the slide, stained and covered with a coverslip. Further development of this technique led to the introduction of permanent air-drying preparations, with this suspension dried on the slide and subsequently stained [17,18]. To enrich the suspension with dividing cells, centrifugation can also be used, especially for smaller objects [19]. The preparations made with this technique yield the best results in terms of chromosome size and shape, and can be stored for years without losing their quality.

Nowadays, routine staining with Giemsa solution and other similar dyes remains a primary technique aimed to visualize parasitoid chromosomes [7]. In turn, routinely stained chromosome sets can be measured to calculate various characteristics of the karyotype. Among these parameters, relative length (RL) and centromeric index (CI) of an individual chromosome are the most important [20]. Both RLs and CIs can be calculated from measurements taken with any regular or specialized software, e.g., KaryoType [21]. Moreover, hymenopteran males lack normal meiosis, but meiotic divisions often can be studied on preparations of developing ovaries (see, e.g., [22]). Furthermore, data on karyotype structure of parasitic wasps also can be complemented by those on the genome size [23,24]. At present, the latter parameter is usually evaluated via flow cytometry [25].

As noted before [8,26], methods of differential staining of parasitoid karyotypes can be provisionally subdivided into “traditional” and “modern” ones. Specifically, “traditional” techniques include C-, AgNOR-, and sometimes also G-banding [27,28,29]. The first two banding methods visualize constitutive heterochromatin and nucleolus organizing regions (NORs), respectively, through the application of alkaline solutions and silver impregnation. In addition, G-bands appear after treating chromosomes with proteolytic enzymes.

“Modern” techniques of differential chromosome staining include the use of base-specific fluorochromes, fluorescence in situ hybridization (FISH) and immunocytochemistry [8,26]. Certain base-specific fluorescent dyes, for example, 4′, 6-diamidino-2-phenylindole (DAPI) and Hoechst 33258, stain AT-rich chromosome segments, whereas chromomycin A_3_ (CMA_3_) reveals GC-rich ones [30]. Moreover, a few other fluorochromes, e.g., propidium iodide (PI), can bind to total DNA irrespective of its base content. Nowadays, FISH represents a powerful tool for physical mapping of various DNA sequences (probes) [31,32]. This technique essentially implies the hybridization of fluorochrome-conjugated DNA probes to metaphase chromosomes or interphase nuclei [33]. These probes can be obtained using different procedures, i.e., via direct synthesis, amplification of existing sequences, or chromosome microdissection [32]. DNA from microdissected chromosomes and/or chromosomal segments can be used to produce chromosome painting [32,33]. Finally, various compounds can be visualized on chromosomes using immunocytochemical techniques [34], which involve specific antibodies conjugated with fluorescent dyes.

## 3. Principal Results

### 3.1. Genetic Features

**Figure 1:**
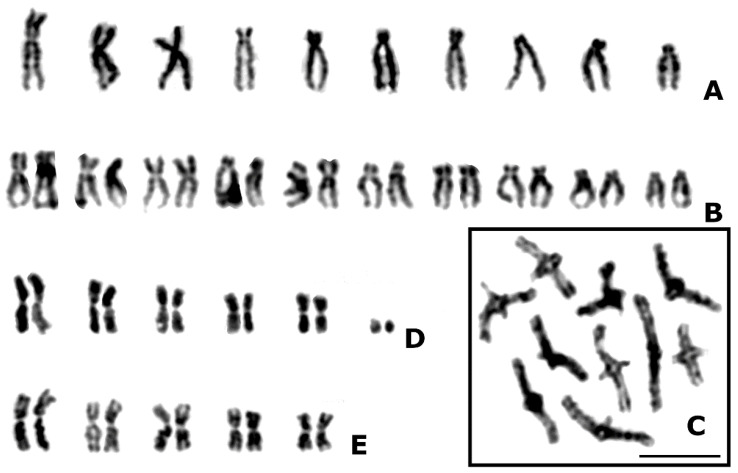
Chromosomes of parasitic wasps; *Eurytoma strigifrons* (Eurytomidae, (**A**–**C**)): male mitotic karyotype, *n* = 10 (**A**); female mitotic karyotype, 2*n* = 20 (**B**); female meiosis (diplotene), *n* = 10 (**C**); *Pronotalia trypetae* (Eulophidae), female mitotic karyotype, 2*n* = 12 (**D**); *Pteromalus* sp. aff. *albipennis* (Pteromalidae), female mitotic karyotype, 2*n* = 10 (**E**). Scale bar = 5 µm.

Arrhenotokous parthenogenesis, i.e., development of male individuals from unfertilized eggs, is characteristic of most members of the order Hymenoptera, including parasitoids [7,35]. In this order, arrhenotoky is usually combined with haplodiploidy, when females and males develop from single diploid and haploid cells, respectively [10,35] (Figure 1A,B). Specifically, haploid males are normally produced under complementary sex determination (CSD), which is an ancestral trait in the Hymenoptera, or by genomic imprinting ([36] and references therein). Nevertheless, diploid or even triploid males can result from intensive inbreeding under CSD [37]. On the other hand, transitions to thelytokous parthenogenesis, i.e., development of females from unfertilized eggs, are relatively frequent in the order [10,35]. Moreover, many members of the family Cynipidae are characterized by cyclical thelytoky, which alternates with arrhenotoky within different generations. In parasitoid Hymenoptera, thelytoky is often (but not always) caused by endosymbiotic microorganisms, which mostly belong to the genus *Wolbachia* [36]. In addition, CSD can be replaced by paternal genome elimination (PGE) in certain populations of a few parasitic wasp species. In most of these populations, the paternal genome is eliminated due to certain factors carried by specific elements, i.e., PSR (paternal-sex-ratio), or parasitic B chromosomes (see below).

### 3.2. Main Details of Karyotype Structure

To demonstrate certain morphological features of chromosomes of parasitic wasps, a few original micrographs of parasitoid karyotypes are given here (Figure 1). All studied species were collected in several locations in European Russia. Air-drying chromosome preparations were made from cerebral ganglia of prepupae and ovaries of adult females (see [7] for technical details). Chromosomes were stained with Giemsa solution; images were obtained using an optic microscope Zeiss Axioskop 40 FL fitted with a digital camera Axiocam 208 color (Carl Zeiss, Germany).

Chromosomes of parasitoid Hymenoptera are relatively long (about 5 µm on average) and monocentric, i.e., each of them carries a single centromere [7,10]. In this group, the haploid chromosome number (*n*) ranges from 3 to 23 [8]. To be precise, 2*n* = 4 (i.e., *n* = 2) was reported for a particular species of the family Trichogrammatidae (see [7]), but this report was apparently erroneous. Distribution of parasitic wasp species by the chromosome number is bimodal, with two obvious modes at *n* = 6 and 11. These modes are characteristic of most members of the superfamilies Chalcidoidea and Ichneumonoidea, respectively [7,26]. However, certain lower taxa that belong to these large groups have substantially different chromosome numbers. For example, members of the subfamily Aphidiinae (Braconidae) have *n* = 3–9, with a clear maximum at *n* = 7 [7]. Moreover, many chalcid families can be subdivided into the “low-numbered” and “high-numbered” ones, with modal *n* values of 5–6 and 10–11, respectively [26].

In many parasitoid karyotypes, chromosomes more or less gradually decrease in size, but strong differences in this respect were found, e.g., in most members of the families Eulophidae and Torymidae [38] (Figure 1D). Chromosomes of many parasitoids are predominantly represented by metacentrics and/or submetacentrics, i.e., they are clearly biarmed [39] (Figure 1D,E). Nevertheless, subtelocentrics and acrocentrics can prevail in the karyotypes of certain Cynipoidea and Chalcidoidea [26,40]. Moreover, both sizes and centromere positions of chromosomes within a given set can be evaluated using the terminology of symmetrical vs. asymmetrical karyotypes (see [21]). Specifically, symmetrical chromosome sets contain obviously biarmed elements (i.e., metacentrics and/or submetacentrics) which show a continuous gradation in length. The more the karyotype deviates from this pattern, the more asymmetrical it is considered.

It is well known that, in addition to A chromosomes, i.e., constant elements of the normal karyotype, B chromosomes, which facultatively present in the chromosome set, can also exist [35]. The highest number of the latter elements was found in a particular parasitoid of the family Eulophidae, *Pnigalio gyamiensis* with 2*n* = 12 + 0–6B [41], and B chromosomes were also detected in some other members of this genus [42]. In many organisms, these chromosomes do not have strong visible effect on external morphology of their carriers (see e.g., [35]), but this is often not the case in parasitoid Hymenoptera. Specifically, particular B chromosomes were discovered in two unrelated members of the superfamily Chalcidoidea, namely, *Nasonia vitripennis* (Pteromalidae) [43] and *Trichogramma kaykai* (Trichogrammatidae) [44,45]. In both these parasitoids, a paternally inherited B chromosome carries a specific factor, which almost completely eliminates the paternal chromosome set (except itself) from the diploid zygote. The latter therefore transforms into a haploid one, which develops into a male (see above). Somewhat analogous elements were also found in *Aphidius ervi* (Braconidae) [46]. However, in this species a pair of B chromosomes is present in an obviously thelytokous strain, and is apparently responsible for a possible doubling of the initially haploid chromosome set of the developing egg.

As noted above, hymenopteran males lack the reductional meiotic division [10]. However, in females of parasitic wasps, normal meiosis occurs in the way similar to many other organisms. In addition, numerous transitions to thelytoky via several basic mechanisms occur in this group (see above). Up to now, morphological diversity of meiotic chromosomes of parasitoid Hymenoptera has been fragmentarily studied ([7] and references therein). Nevertheless, the number of chiasmata per bivalent usually ranges from one to two (Figure 1C). The majority of chiasmata are terminal, but subterminal and even interstitial chiasmata are detected in some cases. If both mitotic and meiotic divisions are available for same species, the number of ring vs. rod meiotic bivalents usually matches that of obviously biarmed chromosomes vs. subtelocentrics plus acrocentrics (see e.g., [22]).

According to the Animal Genome Size Database [47], the genome size of parasitic wasps varies almost by the order of magnitude, i.e., from 0.10 to 0.99 pg. In many cases, it is difficult to detect a substantial pattern of change in this parameter at higher taxonomic levels. Nevertheless, a few comparative studies of closely related parasitoid species, with their chromosome measurements complemented with genome size estimates, were performed during recent years [23,24]. These studies demonstrate that the total length of chromosomes of the haploid karyotype generally increases together with the amount of nuclear DNA. On the other hand, the genome size can change irrespective of visible chromosomal rearrangements, as it was shown for several groups of the genus *Aphelinus* (Aphelinidae) [24]. However, the above-mentioned studies suggest that closely related parasitoid species with karyotypes containing higher proportions of subtelocentric and/or acrocentric chromosomes often have lower genome sizes. If this is the case, amplification or deletion of heterochromatin, which is usually enriched with repetitive DNA sequences, could probably account for this phenomenon.

In parasitoid Hymenoptera, heterochromatic segments are usually restricted to pericentromeric regions [7]. Nevertheless, telomeric and intercalary heterochromatic blocks also occur on chromosomes of these insects [48]. Moreover, shorter arms of certain biarmed chromosomes are fully heterochromatic [49]. In fact, even closely related species of parasitic wasps can strongly differ in the localization and size of heterochromatic segments. For example, chromosomes of *Dirophanes callopus* and *D. invisor* (Ichneumonidae) with 2*n* = 18 and 20, respectively, share similar karyotypes in terms of heterochromatin distribution [49]. Predominantly pericentromeric segments of constitutive heterochromatin are characteristic of both these species. On the contrary, the karyotype of *D. fulvitarsis*, which also has 2*n* = 20, carries extensive heterochromatic segments which also include fully heterochromatic shorter arms of several metacentrics. In addition, polymorphism that involved size of pericentromeric heterochromatin was discovered in a particular pair of metacentric chromosomes of *D. invisor* [49]. Mostly heterochromatic B chromosomes were also found in *A. ervi* ([46], see above).

Among other important features of parasitoid karyotypes, the number and localization of NORs were studied during the last few decades ([42,44,45,50,51,52] etc.). However, these structures can be revealed on chromosomes using different techniques. Historically, NORs on parasitoid chromosomes were first visualized using silver impregnation (AgNOR-banding) (see e.g., [42,44]). This method was able to reveal interspecific differences among closely related parasitoid species [48,53,54]. CMA_3_ is also used to visualize NORs on parasitoid chromosomes ([55,56] etc.), but this fluorochrome sometimes stains multiple CG-rich chromosome segments, which obviously do not represent NORs, as, for example, in *Trichospilus diatraeae* (Eulophidae) [57]. Nevertheless, FISH with rDNA probes can apparently be considered the most reliable technique for revealing these sites ([51,52,55] etc.). All these data suggest that haploid karyotypes carrying one or two NORs, which is characteristic of all studied Cynipoidea and Chalcidoidea, are the most frequent among parasitoid Hymenoptera in general [7], but three and even six rDNA clusters were found in certain Ichneumonidae (*Ichneumon amphibolus*) and Braconidae (*Diachasmimorpha longicaudata*) with higher chromosome numbers (2*n* = 24 and 40, respectively) [50,51].

Chromosomes of quite a few species of parasitic wasps, i.e., *Encarsia berlesei* and *E. inaron* (Aphelinidae), as well as *N. vitripennis*, were studied using G-banding [19,48,58]. In all these parasitoids, G-banding presumably allowed for a precise identification of every chromosome within a given karyotype. However, establishing interspecific homeology between these elements in both members of *Encarsia* appeared impossible.

In parasitoid Hymenoptera, FISH is also used to physically map other repetitive DNA sequences. For example, C_0_t-50 and *Eco*RI repeat were mapped on the chromosomes of *T. kaykai* [44]. In the haploid karyotype of this species, the latter repeat forms two narrow terminal clusters, whereas the C_0_t-50 probe provided strong and predominantly expanded signals on three particular A chromosomes, together with the PSR element. Analogously, a number of interspersed repeats associated with the PSR chromosome were revealed in the karyotype of *N. vitripennis* ([59], also see above). In addition, probes containing different fragments of the genome of a symbiotic polydnavirus mapped a particular site on the chromosomes of *Cotesia congregata* (Braconidae) using non-fluorescence in situ hybridization [60].

The structure of telomeres in parasitic wasps apparently deserves special attention. Previously, the canonical insect telomeric repeat, TTAGG, which was found at least in the lower Hymenoptera [61], was detected neither in parasitoids [51] nor in many Aculeata, except for the families Formicidae and Apidae [62]. However, FISH with the TTATTGGG probe recently visualized this motif at all telomeres of *N. vitripennis* [63]. A subsequent bioinformatic analysis [64] confirmed that the TTATTGGG telomeric repeat (with a few minor changes) is characteristic of all studied members of the superfamily Chalcidoidea. Moreover, an extensive search [65] revealed much broader variation of telomere structure in the order Hymenoptera and in parasitoids in particular. For example, all three examined species of the family Ichneumonidae have extremely divergent telomeric motifs, i.e., TTCCTC in *Buathra laborator*, TTAAAACGCC in *Ichneumon xanthorius*, and a poly-T sequence of 306 to 629 bp in *Amblyteles armatorius* [65].

At present, the only immunochemical study of parasitoid chromosomes was performed a few years ago [55]. In this work, specific antibodies against 5-methylcytosine were used to visualize DNA methylation profiles along the chromosomes of two closely related members of the genus *Entedon* (Eulophidae). In these species, most intensively methylated regions were generally restricted to telomeric segments of all chromosomes [55].

### 3.3. Chromosomal Rearrangements

Although known chromosomal mutations of parasitic wasps were reviewed more than a decade ago in the corresponding monograph [7], new important information on this subject was obtained during the last years. Specifically, chromosome sets of most members of the *Aphelinus varipes* species complex with *n* = 4 contain two metacentrics and two acrocentrics, but the karyotype of *A. kurdjumovi* harbors the only metacentric and three acrocentric chromosomes [24]. In addition, the smaller metacentric of another species, *A. hordei*, has a significantly lower CI if compared to most other members of this complex. Since this chromosome is acrocentric in *A. kurdjumovi*, a sister species to *A. hordei*, we therefore suggested that these two parasitoids share a certain pericentric inversion, followed by another rearrangement of this kind in *A. kurdjumovi* [24]. Together with inversions, other structural rearrangements of parasitoid chromosomes apparently include changes in the amount and localization of the constitutive heterochromatin ([48,49], see above). Finally, translocations are also involved in the process of karyotype transformation of parasitic wasps (see [7] for review).

On the other hand, numerical changes of parasitoid chromosome sets are characteristic of the karyotype evolution of this group. Among these rearrangements, chromosomal fusions are likely to play the leading role [7]. For example, most studied species of the genus *Eurytoma* (Chalcidoidea, Eurytomidae) have *n* = 10 (Figure 1A–C), but apparently related members of two particular species groups, *E. robusta*, *E. serratulae* and *E. compressa*, have *n* = 7, 6 and 5, respectively, due to consecutive pairwise fusions [22]. Analogous rearrangements are likely responsible for the origin of karyotypes with lower chromosome numbers in certain species of another genus, *Metaphycus* (Encyrtidae), i.e., *M. angustifrons* and *M. stanleyi* with *n* = 9 and 5, respectively, as opposed to *n* = 10 in both *M. flavus* and *M. luteolus* [66]. Analogously, the chromosome set of *Andricus mukaigawae* (Cynipidae) with 2*n* = 12 apparently results from several centric fusions occurred in the karyotype having 2*n* = 20, which predominates in the genus *Andricus*, and another rearrangement of this kind can be observed in *A. kashiwaphilus* with 2*n* = 10, a sister species to *A. mukaigawae* [18]. Similarly, the chromosome set of *Tycherus ischiomelinus* (Ichneumonidae) with 2*n* = 18 likely originates via at least two consecutive fusions from that characteristic of most other members of this genus (2*n* = 22), including its putative sister species, *T. australogeminus* [67].

However, many details of chromosomal fusions in parasitoids remain poorly known. For instance, a molecular study of the *Lariophagus distinguendus* species complex (Pteromalidae) suggests a particular fusion in the karyotype with *n* = 5, as opposed to the chromosome set with *n* = 6 [68], and even sophisticated techniques of chromosomal analysis cannot fully resolve all rearrangements occurring in this complex [69]. Specifically, a combination of microdissection with chromosome painting demonstrated that the only acrocentric and a smaller metacentric chromosome in the karyotype with *n* = 6, respectively, correspond to the shorter and longer arms of the largest metacentric in the chromosome set with *n* = 5. Nevertheless, we still do not know whether this fusion was preceded by a pericentric inversion and, consequently, whether this rearrangement was either centric or tandem [69]. In the former case, the inverted section of the chromosome can represent a “supergene” [70], i.e., a non-recombining chromosome segment already discovered e.g., in some ants [71]. Chromosome painting was also used to distinguish between morphologically similar metacentrics in the haploid karyotype of *N. vitripennis* with *n* = 5 [58].

Putative fissions and other numerical changes of parasitoid karyotypes also remain poorly studied [7]. Nevertheless, obvious chromosomal mutations of that kind were recorded in these insects. Among these rearrangements, polyploidy was discovered in the only fully triploid thelytokous species of parasitic wasps, *Diplolepis eglanteriae* (Cynipidae) [40]. Apart from this record, a polyploid strain (containing both triploid females and diploid males) of *N. vitripennis* is maintained in the lab [72]. However, controlled crossings are needed to reliably obtain polyploid individuals in this case. Diploid males were also discovered in a number of other parasitoid species, especially those having CSD (see above). In addition, low fecundity of triploid females of *N. vitripennis* was apparently ascribed to frequent aneuploidy (and hence high lethality) in the offspring, but no cytological evidence, which could favor this assumption, was presented [72]. On the other hand, viable aneuploid individuals were recorded in a few other species of parasitic wasps [7], e.g., *Torymus bedeguaris* [51]. Finally, intraspecific changes in the number of B chromosomes were also observed in some parasitoids ([42], also see above).

### 3.4. Karyotype Evolution

**Figure 2:**
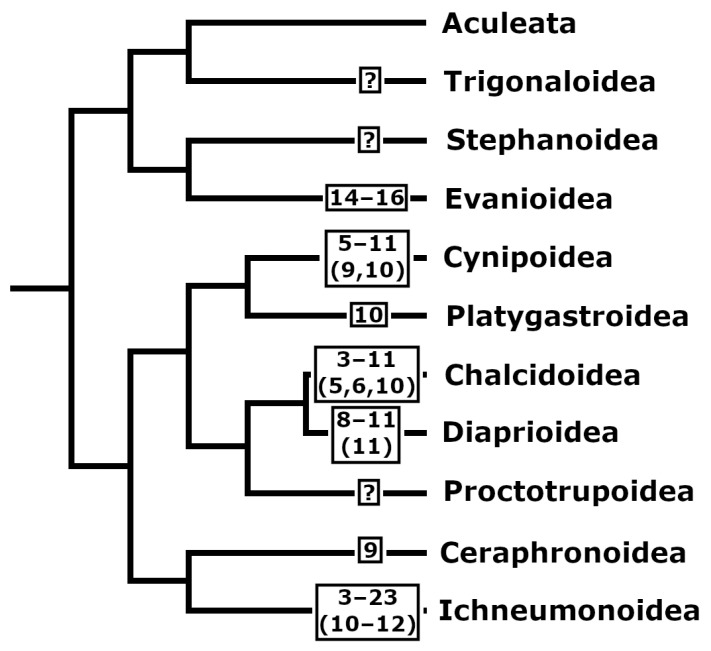
Phylogenetic tree of parasitoid superfamilies (simplified from [73]). Characteristic ranges of haploid chromosome numbers are shown for each group; most frequent *n* values are given in parentheses.

Phylogenetic analysis of chromosomal variation among higher taxa of the order Hymenoptera suggests that ancestral parasitoid karyotypes apparently had relatively high *n* values (*n* = 14–17) with the predominance of biarmed chromosomes [7] (Figure 2). In turn, independent multiple reductions in the chromosome number occurred within various lineages of parasitic wasps [39] (see, e.g., Figure 1B,D,E). In fact, two main processes dominated during the karyotype evolution of parasitoid Hymenoptera. In addition to the reduction in the chromosome number, it is karyotypic dissymmetrization due to an increase in size differentiation of chromosomes as well as in the proportion of subtelocentrics and acrocentrics within karyotypes [7]. However, reverse processes, i.e., an increase in the chromosome number and/or in the proportion of metacentrics and submetacentrics within certain karyotypes, occurred within various lineages, but these processes were far more restricted (see e.g., [22]).

As far as taxonomic levels of karyotype evolution of parasitic wasps are concerned, we can provisionally identify chromosomal changes below the species level as well as within higher taxa. In parasitoids, intraspecific chromosomal variation, either as spontaneous mutations or in the form of stable polymorphism, includes differences in the amount of the constitutive heterochromatin, polyploidy, aneuploidy and changes in the number of B chromosomes [7]. Similar differences in the localization and amount of the constitutive heterochromatin and those in the number and localization of NORs are also characteristic of groups of closely related species, together with inversions and chromosomal fusions. This is apparently true for the higher taxa as well (also see above). Another interesting question regards possible symmetry and/or asymmetry of the macroevolutionary change, e.g., during the increase/decrease in the chromosome number. At present, it seems that these changes are asymmetrical in terms of mutations involved in these processes [7], but this problem is definitely far from being fully resolved.

If the genome size is also taken into account, the corresponding analysis demonstrates that the karyotype structure often evolves independently of it [24]. In turn, this can be explained either by the possibility of accumulation/loss of repetitive sequences without changing the visible karyotype structure, or by the fact that many chromosomal rearrangements of parasitoid karyotypes, e.g., fusions/fissions, do not significantly alter the genome size.

### 3.5. Taxonomic Implications

Nowadays, there is rising awareness among the experts about the significance of chromosomal data for parasitoid taxonomy [9]. This is mainly due to the very nature of karyotypic characters, which often can distinguish between closely related forms. In addition, this situation can be partly explained by understanding of the complex structure of many morphospecies, which, in turn, was realized mainly due to molecular studies. Nevertheless, karyotypic research also demonstrates that these morphospecies often include certain cryptic lineages.

Perhaps the most interesting case, which shows the importance of the chromosome study for parasitoid taxonomy, can be found in the family Pteromalidae. Specifically, karyotypic research of the presumably well-known cosmopolitan parasitoid of a wide array of coleopteran-stored product pests, *Anisopteromalus calandrae*, discovered two different chromosome sets with *n* = 5 and 7 [74]. A subsequent study demonstrated that these karyotypes belong to two different species, which have substantial morphological differences, alternative life-history strategies and are reproductively isolated from each other [74,75,76]. Moreover, the species with *n* = 5 appeared to be new to science and was later described as *Anisopteromalus quinarius* [77]. Another similar example involves two cryptic species of the *L. distinguendus* complex with *n* = 5 and 6, which belong to the same family [68,69].

Analogously, karyotypic characters helped reveal previously unknown species in other chalcid families. For example, both newly described *Eupelmus barai* and *E. rameli* (Eupelmidae) with 2*n* = 12 differ from two most closely related species, *E. vesicularis* and *E. messene*, respectively (both have 2*n* = 10), in a combination of morphological and chromosomal characters [78]. Furthermore, *E. vladimiri* also has 2*n* = 10, but, apart from all other studied members of the subgenera *Macroneura* and *Eupelmus* s.str., its karyotype contains five pairs of metacentric chromosomes [78]. In the family Aphelinidae, chromosome sets with 2*n* = 10 were discovered in two different populations formally belonging to *Encarsia sophia*, but these karyotypes differ in some morphometric features as well as in the localization of NORs [54]. However, no new species was formally described in the latter case.

New cryptic species were also discovered in the Ichneumonidae [66]. For example, *T. australogeminus* with 2*n* = 22 was initially distinguished from closely related *T. ischiomelinus* with 2*n* = 18 on the basis of their karyotypic features. Moreover, *Aethecerus ranini* and *A. dispar* differ in their chromosome numbers as well (2*n* = 22 and 24, respectively). In addition, a new member of the genus *Andricus*, *A. kashiwaphilus* (Cynipidae), was detected due to the karyotypic differences between this species and *A. mukaigawae* (see above) [18].

Furthermore, the presence of cryptic species is suggested in a few other cases (see [7] for review). However, different chromosome numbers reported for same parasitoid species could also point to erroneous taxonomic identifications.

## 4. Conclusions

The above-mentioned results demonstrate that comparative cytogenetics of parasitoids currently represents an intensely developing research field, with many interesting data obtained during recent years. Most of these results can be used to better characterize certain taxa of parasitic wasps and/or to distinguish between them. Nevertheless, possible areas of future progress of the comparative cytogenetic research of parasitoids also can be outlined. First, chromosome study can become an effective tool of practical distinction between different species of economically important parasitic wasps. For example, karyotypes of all examined members of the genus *Trichogramma* with the same chromosome number, *n* = 5, once believed to be virtually identical [14], now appear to have substantial morphometric differences [26,57,79,80]. Moreover, recent data obtained from massive genome sequencing suggest that chromosome morphometry can be used for a precise evaluation of the degree of completeness of the genome assemblage [81]. On the other hand, different FISH probes, including those obtained by chromosome microdissection, will undoubtedly be used for physical mapping of DNA sequences. Finally, studies of the genome size, meiotic chromosomes as well as comparative immunocytochemistry will further highlight evolutionary transformations of parasitoid karyotypes. All these studies will certainly lead to a better understanding of the genome evolution of parasitic wasps.

## Data Availability

All relevant data are within the present paper.
